# The Effect of MitoQ on Aging-Related Biomarkers: A Systematic Review and Meta-Analysis

**DOI:** 10.1155/2018/8575263

**Published:** 2018-07-12

**Authors:** Andrea J. Braakhuis, Rohith Nagulan, Vaughan Somerville

**Affiliations:** Faculty of Medical & Health Sciences, Discipline of Nutrition, The University of Auckland, Private Bag 92019, Auckland, New Zealand

## Abstract

Mitochondria are metabolically active organelles that produce significant reactive oxygen species, linked with aging and degenerative diseases. In recent years, particular focus has been put on mitochondria-targeted antioxidants, to decrease the concentration of reactive oxygen species and help alleviate the accumulation of oxidative damage and associated aging. MitoQ is a mitochondria-targeted antioxidant of which is reported to support healthy aging. The aim of this systematic review is to investigate the effects of MitoQ on oxidative outcomes related to the aging process. A predeveloped search strategy was run against MEDLINE (Ovid), EMBASE (Ovid), and CINAHL databases, which identified 10,255 articles of interest, with 27 of these finalised for use after screening. Three outcomes had sufficient data to meta-analyse nitrotyrosine concentration (190 animals, SMD −0.67, 95% CI (−1.30, −0.05), *p* = 0.04), membrane potential (63 animals, MD 11.44, 95% CI (1.28–21.60), *p* = 0.03), and protein carbonyl concentration (182 animals, SMD −0.13, 95% CI (−0.44, 0.18), *p* = 0.41). MitoQ intervention produced a statistically significant reduction in nitrotyrosine concentration and increased membrane potential. MitoQ may be of some benefit in alleviating oxidative stress related to aging.

## 1. Introduction

Aging, an inevitable biological process, is characterised by a general decline in physiological function that leads to morbidity and mortality. Specific causes of this decline are still uncertain, although various lines of evidence implicate oxidative damage and mitochondrial decline as being a fundamental driving force behind this process [1]. Sustained damage inflicted by endogenously produced oxidants is the likely cause of age-related deficits in mitochondrial function and general physiological decline common to all aging organisms [1].

The postulated relationship between cellular decline and reactive oxygen species (ROS) has been well explored in the free radical theory of aging, which suggests that human lifespan and degenerative disease are tied to the adverse effects of ROS on cell structure and function [2]. Once produced, ROS react with lipids, proteins, and nucleic acids causing oxidative damage to these macromolecules, over time contributing to the aging process [3]. Various biomarkers have been used to measure the effects of oxidative damage in nutraceutical research, including lipid peroxidation, antioxidant activity assays, antioxidant enzyme activity, and DNA damage [2, 4, 5]. 7,8-Dihydro-8-oxo-deoxyguanosine (8-oxo-dG) is one of the most abundant and well-characterised DNA lesions caused by ROS [3].

Mitochondria are among the most metabolically active organelles in the body and are a primary source of energy production and oxidative phosphorylation [6]. Oxidative phosphorylation, a process in energy production, results in the production of ROS. The free radical superoxide in particular is created in the mitochondria through nonenzymatic means when leakage of electrons from various complexes occurs and subsequently binds to oxygen molecules [7]. Other ROS markers are of interest as well in the mitochondria. Malondialdehyde (MDA) is formed as a product of lipid degradation and is mutagenic in nature due to its ability to create DNA adducts. It is also a mitochondrial toxin, inhibiting respiration and enzyme catalytics [8]. As both the major producer and primary target of ROS, mitochondria are thought to play an important role in aging.

Nitrotyrosine (3-NT) is a biomarker of protein oxidation produced upon the nitration of protein residues, which alters protein structure and function [9]. In aging-related oxidative stress, peroxynitrite production, resulting from the reaction between nitric oxide and superoxide, could increase. Peroxynitrite is responsible for nitration of tyrosine residues in proteins. Therefore, the presence of nitrotyrosine in plasma proteins is considered an indirect evidence of peroxynitrite production and an indicative of oxidative stress [9]. Protein carbonyls arise from lipid peroxidation and are associated with many chronic human illnesses [10]. Mitochondrial membrane potential is also an important factor to consider as it affects energy production as well as organelle turnover and elimination [11]. Membrane potential has been used as a proxy marker for mitochondrial function, which naturally declines with age [3].

Generally speaking, decreasing the concentration of ROS and thereby potential damaging capabilities, it is hypothesised that the aging process can be delayed. This concept has inspired a host of nutraceuticals aimed at alleviating oxidative damage, particularly in the mitochondria [12].

To decrease mitochondrial oxidative damage, a number of mitochondria-targeted antioxidants have been developed. One such mitochondria-targeted antioxidant is MitoQ, which consists of a quinone moiety linked to a triphenylphosphonium (TPP) moiety by a 10-carbon alkyl chain [13]. Currently, the effect of MitoQ on oxidative stress is inconclusive and predominately researched in studies with small participant numbers [13]. Therefore, the purpose of this systematic review is to investigate the effect of MitoQ on markers of oxidative stress and aging, which could form the basis of future human clinical trials.

## 2. Material and Methods

### 2.1. Search Strategy and Selection Criteria

A literature search investigating the effect of MitoQ on oxidative stress-related aging markers in clinical and preclinical trials was conducted using a created search strategy. The search strategy included the following concepts: MitoQ and quinone derivatives, oxidative stress markers, and mitochondria, with terms optimised for each database using Boolean operators. Data were collected from the following databases: MEDLINE (Ovid), CINAHL, and EMBASE (Ovid). The databases AMED and EMBASE classic were also considered but were excluded due to AMED having an absence of MitoQ-relevant articles and EMBASE classic cataloguing articles from 1947–1973 during which MitoQ had not been synthesised. The strategy employed the use of medical subject headings (MeSH terms) where possible, but in circumstances when a MeSH term was invalid in a particular database, an equivalent term was substituted. Articles obtained from the search were then screened by title and abstract, and duplicates were removed. The 247 full-text articles that remained were then screened by two authors (VS and RN) independently based on a predefined inclusion and exclusion criteria. The inclusion criteria were as follows:

1. Animal or human studies
2. Placebo or control intervention
3. Crossover or parallel in design
4. Randomised controlled trial (RCT)
5. Studies that measure lipid peroxidation, oxidative damage, or one of the secondary outcome measures

**Figure 1:**
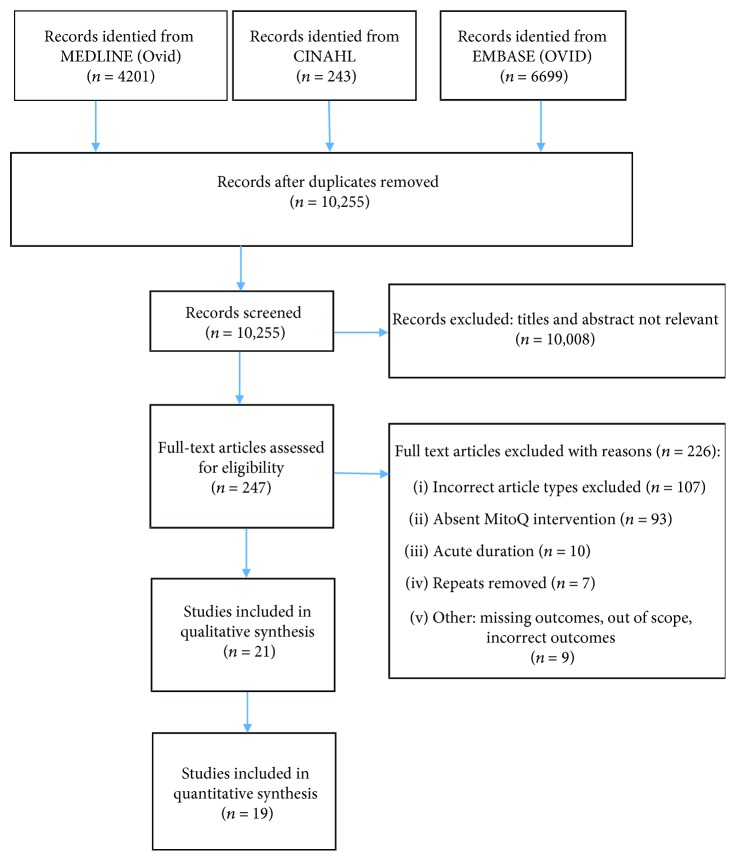
PRISMA chart.

Studies were excluded if cell based, lacked inferential statistics (standard error (SE)/standard deviation (SD) or *p* values), or if data could not be accurately extracted. An article was determined to be included or excluded if both authors achieved consensus. In cases of disagreement or if either author was unsure of the article's status, a third author (AB) made a final decision. After this, 18 articles were included for data analysis (see Figure 1). The intended outcomes were as follows:

1. Primary outcomes
  - Lipid peroxidation (malondialdehyde (MDA), thiobarbituric acid reactive substance (TBARS), rinary 8-epi-prostaglandin-F2*α* (8-epi-PGF2*α*), and urinary 8-hydroxydeooxyguanosine (8-OHdG)
  - Oxidative damage (DNA/RNA damage)
2. Secondary outcomes
  - Protein carbonyl concentration
  - Antioxidant enzymes (catalase, superoxide dismutase, and glutathione peroxidase activity or concentration)
  - Mitochondrial membrane potential
  - Antioxidant activity (FRAP and ORAC)
  - 3-Nitrotyrosine
  - Nitric oxide concentration
3. Genetic-related outcomes
  - Genes related to oxidative stress response and/or antioxidant activity (Nrf2, Hmox1, Sepp1, and Srxn1)
  - Reactive oxygen species metabolism (Fmo2, Sod2, and Ucp2)

## 3. Data Collection and Assessment of Bias

Data was independently collected by two authors (VS and RN). Where data was presented in graphical format, Plot Digitizer software was used to accurately extract data (both authors individually extracted the mean and respective inferential statistics, and if there was a difference, the average value of the two was used) (Source Forge, GNU Library License version 2.0) to confirm the visually collected data. Where studies provided a range of characteristics (i.e., 150–200 g), the middle value was used. Conversely, if studies provided a range for sample size, the lower number was utilised to prevent overpowering. For control data, the authors extracted the values for the closest possible variable; for example, when control, high-fat diet, and high-fat diet + MitoQ was used, high-fat diet was used as “control.” If data was presented as median with interquartile range, the corresponding author for the paper was contacted for the mean and SD. One author [14] was contacted and provided the mean and SD results.

The review of abstracts and full texts retrieved followed the Preferred Reporting Items for Systematic Reviews and Meta-Analyses guidelines (PRISMA) [15]. Collected data included characteristics of participants and study, intervention type and dose, study description/overview/setting, study recruitment, risk of bias, and outcomes.

With the use of the following 6 categories, 2 reviewers (RN and VS) completed the assessment of the risk of bias for each study independently:

1. Sequence generation
2. Allocation concealment
3. Blinding
4. Missing outcome data
5. Risk of reporting bias
6. Other sources of bias according to SYRCLE's risk of bias tool for animal studies [15]

### 3.1. Data Synthesis

Literatures reporting the standard error of the mean (SEM) were converted to SD prior to analysis by multiplying by the square root of the sample size. Where a result was presented in relation to control, and the control lacked inferential information, the same SD for the invention was used for control. The units of MitoQ dosage varied between studies, so to improve clarity of interpretation, all doses were converted into a common metric (mg·day^−1^). Ten studies [16–25] reported MitoQ concentration in moles, and so the molar mass of the supplement active ingredient (680 g·mol^−1^) was used to convert this value into mg·day^−1^. The remaining studies [14, 26–32] reported dosage per kg weight; in this case, the average subject weight was used to convert this value into mg·day^−1^.

### 3.2. Data Analysis

For outcome measures with data from three or more publications, a meta-analysis was performed with the Review Manager software (version 5.3). When outcomes were measured in different units, the study effect size was calculated from standardised mean differences. Alternatively, when an outcome was measured in identical units, mean difference was utilised. When pooling data, the fixed effect model was adopted, except when the heterogeneity score (Higgins score) was greater than 60%, the random effect model, with other factors in agreement with the model type [15]. The subject number from research using the same participant was artificially lowered when analysed to reduce the effect from the same subject, as was conducted in the membrane potential meta-analysis [27].

## 4. Results

### 4.1. Study Selection

After the individual screening of the 10,255 articles, the articles with title/abstracts not pertaining to the review were deleted with 247 articles remaining. Two reviewers (VS and RN) assessed the remaining articles, and 27 of these were included after mutual consensus. The PRISMA diagram outlining the selection process, including the number of studies at each stage and reasons for exclusion, is presented in Figure 1.

### 4.2. Study Characteristics

The characteristics of studies included in the meta-analysis are shown in Table 1. In summary, all of the studies were RCTs and used a parallel design. The studies were conducted in a range of countries, including seven in the United Kingdom [11, 18–20, 26–28], eight in the United States [16, 17, 21, 22, 29, 33–35], three in France [30, 31, 36], two in India [23, 32], and one in New Zealand [37], Uruguay [24], China [38], and Singapore [25]. A total of 13 animal studies used MitoQ as supplementation, either dissolved in drinking water [16, 17, 19, 20, 32, 35] or added as part of rodent diet [18, 26, 31]. One human study provided MitoQ as dietary supplement [33], and a further study used MitoQ as stock solution [25]. The remaining used a combination of IV infusion [14, 17, 21, 22, 28, 29], to administer MitoQ.

The range of MitoQ dose varied from 0.1–340 mg·day^−1^. Seven studies used a MitoQ dosage below 50 mg·day^−1^ [14, 25–29, 33], seven studies used a MitoQ dosage above 50 but below 300 mg·day^−1^ [16, 20–22, 30–32], and 5 studies used a MitoQ dose above 300 mg·day^−1^[17–19, 23, 24].

The major studies can be subdivided into three durations. Dare et al., Lowes et al., and Powell et al. [14, 28, 29] had an intervention duration of within 24 hrs; nine studies had a duration of 1–8 wk [16, 17, 22, 24–26, 31, 33], and six studies had an intervention duration of 8–28 wk [18–21, 32, 35]. The studies varied in their reported outcomes, although they all measured at least one of the predetermined primary or secondary outcomes.

Six articles included female animals [17, 18, 20, 21, 23, 24], while the remainder used all male subjects. The Sprague-Dawley rat breed was predominantly used as interventional subjects, with 6 articles [14, 23, 26, 27, 30, 31], including them. C57BL/6 mice were the next common breed of animal used, with four articles [16, 19, 20, 28], including them.

Of the 27 studies included, seven studies measured protein carbonyl concentrations, [17–19, 23, 25, 28, 32], six measured 3-NT, [20–23, 26, 35], and three each measured MDA [23, 29, 33] and four membrane potential [27, 30, 31, 38]. The remaining outcome measures were reported by various studies.

### 4.3. 3-Nitrotyrosine

**Figure 2:**
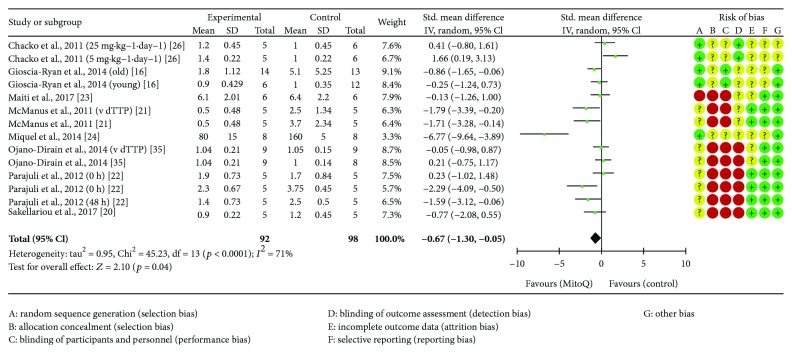
Nitrotyrosine forest plot.

Eight studies reported 3-NT, [16, 20–24, 26, 35], as a result of protein oxidation, totalling 190 animals, with MitoQ intervention (*n* = 92) or control (*n* = 98). The meta-analysis of the change in 3-NT is shown in Figure 2. MitoQ supplementation had a significant reduction in 3-NT concentration (*p* = 0.04).

### 4.4. Membrane Potential

**Figure 3:**
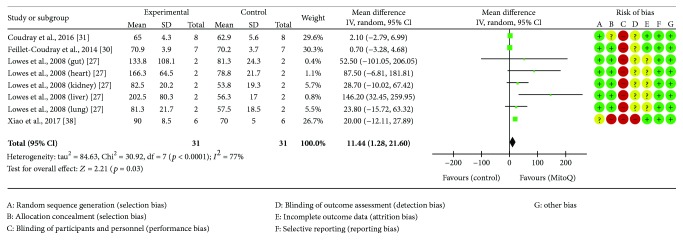
Membrane potential forest plot.

Four studies reported membrane potential, [27, 30, 31, 38], totalling 62 animals, with MitoQ intervention (*n* = 31) or control (*n* = 31). MitoQ significantly increases membrane potential (*p* = 0.03) (see Figure 3).

### 4.5. Protein Carbonyls

**Figure 4:**
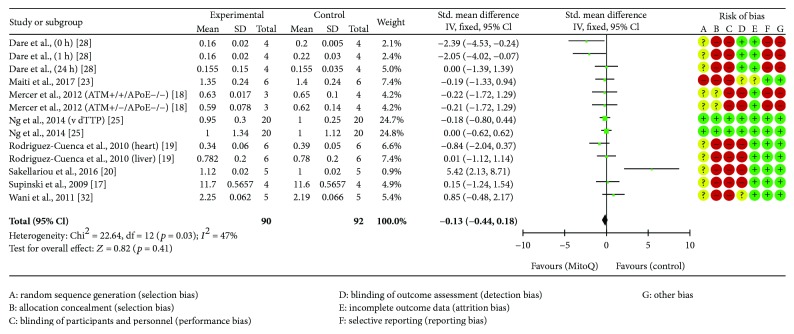
Protein carbonyl forest plot.

Eight RCTs reported protein carbonyl concentration, totalling 182 animals, [17–20, 23, 25, 28, 32]. All participants were given either a MitoQ intervention (*n* = 90) or control (*n* = 92). MitoQ treatment tends to favour decrease of protein carbonyl concentration though the change is statistically insignificant (see Figure 4).

### 4.6. Other Outcomes

The remaining outcomes with insufficient data to meta-analysed are displayed in Table 2. In general, lipid peroxidation biomarkers are lower with MitoQ treatment, with remaining measures unclear. Three studies reported MDA [23, 29, 33], totalling 42 animals/humans, with MitoQ intervention (*n* = 21) or control (*n* = 21). Few studies reported results for gene-related outcomes.

### 4.7. Bias

In summary, four studies reported adequate sequence of allocating participants to treatment [25, 27, 30, 33], whereas the remaining 16 did not state the method of allocation and therefore had unclear bias.

Similarly, two studies reported a satisfactory method of concealing allocation [25, 33], with the remaining categorised as high risk because the method was not reported by the investigators.

All studies were single-blind RCTs except Lowes et al., Ng et al., and Shill et al. [25, 27, 33], which employed a double-blind RCT design. As a consequence, the three double-blind RCTs had a low blinding bias, while the remainder had high or unclear blinding bias. Most studies had zero withdrawals/animal deaths during the study period; therefore, no outcome data were missing. Ojano-Dirain et al. [35] reported that 1-2 mice perished per group yet this did not match the difference, so the study was categorised as unclear. Additionally, Miquel et al. [24] did not provide the exact number of subjects per group and so was categorised as unclear.

The main source of “other bias” resulted from the declaration of funding. Two of the studies were funded by commercial interests, and the arrangement between the researchers and funder was not well defined. [18, 28]. In addition, Mercer et al. [18] declared a conflict of interest also due to holding stock in Antipodean Pharmaceuticals who produces MitoQ. As it was not possible to determine the impact the conflict may have had on these two studies, they were both allocated high risk. The remaining 18 studies were assigned low bias risk as authors had no conflicts of interest to declare and funding appeared to be independent of third parties with vested interest in MitoQ.

## 5. Discussion

This review has examined the effect of MitoQ on oxidative stress markers related to the aging process. Our findings indicate that MitoQ has a statistically significant reduction in concentrations of 3-NT. This is of interest as nitration of protein residues has been shown to inhibit enzyme catalytics [7], and so MitoQ may promote efficiency of cellular processes as well as help decrease the concentration of reactive oxygen species. Of special note is the manganese-dependent superoxide dismutase (MnSOD) protein, which is inhibited by 3-NT and serves as a superoxide scavenger in the mitochondria, preventing formation of the hydroxide ion which is a powerful oxidant. MnSOD upregulation has been linked with an increase in mean lifespan [39] and decrease in endothelial dysfunction with aging [40] in the animal model. This bodes positive effects for human aging; however, the true effect is still unknown for the majority of research has been conducted on animals.

Mitochondrial membrane potential has been shown to significantly increase upon administration of MitoQ, suggesting an upregulation in the functioning capacity of mitochondria with supplementation. Mitochondrial membrane potential is commonly used as an indication of functional status [11]. Membrane potential arises from a proton gradient established across the mitochondrial inner membrane which drives ATP production through oxidative phosphorylation. While decreased membrane potential (depolarization) indicates damaged, dysfunctional mitochondria that cannot meet cellular energy demands, increased membrane potential (hyperpolarization) suggests increased functional capacity and work conducted.

Four articles [27, 30, 31, 38] were found investigating the impact of MitoQ on membrane potential; however, Lowes et al. provided data subcategorised by organ which were all included in the meta-analysis. This is an interesting focus for additional research as Lowes et al. indicate that there is an outcome variation between organs which could indicate that MitoQ mechanism of action has affinity for certain areas in the body, potentially allowing for a targeted response in systemic disease. While the concept of organ affinity by MitoQ has not been well established, it is worthy of further investigation.

Oxidatively modified forms of proteins accumulate during aging, oxidative stress, and in some pathological conditions and have been studied for some time [41]. Protein carbonyl is an oxidative damage marker resulting from the damage of accumulated protein. Protein carbonyl concentration as an outcome of MitoQ was better investigated, with seven articles reporting it. Based on our data, the effect of MitoQ on protein carbonyl is insignificant, with variable results between studies.

MDA is an important DNA mutagen, and it is also a more obscure oxidative outcome and appears not to have been investigated in great detail, with only three studies sourced, two animals, and one human.

Lipid peroxidation biomarkers not meta-analysed were lower when supplementing with MitoQ. Antioxidant defence enzymes, such as superoxide dismutase (SOD), catalase (CAT), and glutathione peroxidase (GPx), are crucial for breaking down the harmful end products of oxidative phosphorylation, and our data suggests that supplementation with MitoQ has little impact on the antioxidant enzyme content and activity. It is useful to consider that provision of MitoQ does not appear to downregulate antioxidant enzyme activity and therefore function is at least maintained. Varied results were seen with the effect of MitoQ on ROS, mitochondrial DNA damage, and oxidative stress-related gene expression.

No significant differences in outcome were noted among the different animal breeds. A range of animal study models were used in the included articles; however, of particular interest, it is the obesogenic model [30, 31] or the high-fat western diet model [18], although no notable difference between oxidative stress models was indicated.

The recommended commercial dosage of MitoQ is 10 mg per day (taken from the manufacturer's website); however, most of the included studies met or exceeded the recommended doses, acknowledging the extrapolation a human recommendation to animal studies is difficult. MitoQ achieves action by means of its TPP cation, which is transported into mitochondrial matrix by means of membrane potential [42]. Once within the mitochondria, it is reduced to ubiquinol by respiratory chain complex II and is now able to provide an antioxidative effect by electron exchange with oxidative species. However, the bioavailability of the molecule must be taken into consideration: as MitoQ is consumed orally and so is subjected to first pass metabolism [43] of the liver and stomach, meaning the quantity of supplement reaching the systemic circulation will be decreased from the full dosage. Bioavailability and delivery to site of action have been established and cited in the literature, at least in vivo [44]; however, delivery of MitoQ to skeletal muscle mitochondria in human participants has not yet been conducted.

It must be noted that all studies supplied MitoQ under conditions of stress; however, this in turn may raise the amount of reactive oxygen species in subjects due to chronic psychoemotional stress [45]. Due to this, ROS concentrations may be influenced depending on the length of study and also the study model, whose characteristics such as animal caging condition, lighting, feeding regularity, and human interactions may contribute to variable degrees of the aforementioned psychoemotional stress. Living conditions of the animal subjects is therefore an important variable of consideration; however, the effect does not appear to be significant as study designs, as reported in the articles, note similar patterns: namely, standard rodent chow, drinking water ad-libitum, and 12-hour light/dark cycle. This model is unlikely to represent the typical consumer of the MitoQ supplement and so raises the question as to whether MitoQ effect would change when applied to the human model.

MitoQ appears to require a high level of dosage to induce toxicity. When administered through intravenous means, animals subject to MitoQ treatment are able to be given up to 20 mg MitoQ/kg without any toxicity [44]. Orally, animals fed with MitoQ dissolved in drinking water at 500 *μ*M (339 mg) had no adverse effects, though toxicity was reported after 10 days of intervention at a dosage of greater than 2 mM (1357 mg) [46]. This indicates that MitoQ is well tolerated and that the commercial dosage of the product is well within the therapeutic range. Interestingly, rats fed with 500 *μ*M MitoQ over a 6-month period were reported to have an accumulation of MitoQ in the heart (113 pmol/g) and liver (20 pmol/g) [44]. Therefore, it appears that MitoQ has promise in targeting ROS production associated with heart failure—though caution would be advisable in those conditions as the effects of MitoQ on cardiac rhythm upon ischemia are unknown.

## 6. Conclusion

This is the first systematic review and meta-analysis of the effects of MitoQ on oxidative stress age-related outcomes. The findings demonstrate that MitoQ intervention may reduce nitrotyrosine concentration and improve mitochondrial function as measured by membrane potential. While protein carbonyl concentrations were lower with MitoQ treatment, the results were insignificant. Animal studies were primarily used in this review, as important preclinical data; however, these results give further basis and direction for human clinical trials.

## Figures and Tables

**Table 1:** Summary of included studies.

Study (ref)	Participants	Study design	Intervention	Outcomes measured
Chacko et al., 2011 [26]	Male Sprague-Dawley rats (260–300 g)	Parallel design, chronic ethanol exposure model, *n* = 11, blinding: single	5 and 25 mg·kg^−1^·day^−1^ (1.3 and 7.5 mg·day^−1^) MitoQ for 4 weeks	Protein oxidation (3-NT), mitochondrial ROS production in liver samples
Coudray et al., 2014 [31]	Male Sprague-Dawley rats (190–200 g), 6 weeks of age	Parallel design, rat feeding model: obesogenic diet, *n* = 16, blinding: single	0.86 g·kg^−1^ (167.7 mg·day^−1^) MitoQ in a high-fat diet for 8 weeks	Antioxidant enzymes (CAT, GPx, SOD), lipid peroxidation (8-isoprostane), mitochondrial membrane potential, genes related to oxidative stress response/antioxidant activity (Nrf2) in liver samples
Dare et al., 2015 [28]	Male C57BL/6 mice	Parallel design, ischemia-reperfusion injury model, *n* = 8, blinding: single	Mice exposed to sham operation with 4 mg·kg^−1^ (0.10 mg·day^−1^) MitoQ administration, for 24 hours reperfusion	Oxidative damage (mtDNA), protein oxidation (PC) in kidney sample
Feillet-Coudray et al., 2016 [30]	Male Sprague-Dawley rats (175–200 g), 6 weeks of age	Parallel design, obesogenic diet-fed, *n* = 15, blinding: single	0.86 g·kg^−1^ (161.25 mg·day^−1^) MitoQ given in high-fat diet for 8 weeks	Mitochondrial ROS production and membrane potential in skeletal muscle (gastrocnemius) sample
Gioscia-Ryan et al., 2014 [16]	Male C57BL/6 mice, 6 or 25 months of age	Parallel design, MitoQ supplemented in drinking water, *n* = 18, blinding: single	250 *μ*M (169.70 mg·day^−1^) MitoQ dissolved in drinking water for 4 weeks	Acute mitochondrial ROS production, protein oxidation (3-NT) in smooth muscle (carotid artery) sample
Lowes et al., 2008 [27]	Male Sprague -Dawley rats (500 g)	Parallel design, LPS/PepG model of sepsis, *n* = 8, blinding: single	7.5 *μ*mol·kg^−1^ (2.54 mg·day^−1^) MitoQ given in 1 ml saline bolus then 5 *μ*mol·kg^−1^·h^−1^ at follow-up for 6 hours	Mitochondrial ROS production, mitochondrial membrane potential change in lung, liver and gut, and heart samples
Lowes et al., 2013 [14]	Male Sprague-Dawley rats (463 g)	Parallel design, at LPS/PepG model of sepsis, *n* = 10, blinding: double	1.5 *μ*mol·kg^−1^ (0.69 mg·day^−1^) MitoQ given in 1 ml bolus saline with 1 *μ*mol·kg^−1^·h^−1^ as follow-up for 5 hours	Protein oxidation (PC), oxidative damage (DNA) in liver sample. Median data are reported.
Maiti et al., 2017 [23]	Male and female adult Sprague-Dawley rats (210 ± 18.19 g)	Parallel design, Pb neurotoxicity model, *n* = 12, blinding: single	500 *μ*M (339.41 mg·day^−1^) MitoQ dissolved in drinking water for 15 days	Lipid peroxidation (MDA, TBARS), protein oxidation (PC, 3-NT), mitochondrial membrane potential in brain sample
McManus et al., 2011 [21]	Female 3xTg-AD mice (transgenic and wild-type)	Parallel design, transgenic mouse model of Alzheimer's disease, *n* = 10, blinding: single	100 *μ*M (67.88 mg·day^−1^) MitoQ dissolved in drinking water for 5 months	Lipid peroxidation (TBARS), protein oxidation (3-NT) in cultured brain samples
Mercer et al., 2012 [18]	Male and female ATM^+/−^/ApoE^−/−^ and littermate ATM^+/+^/ApoE^−/−^ mice, 6 weeks of age	Parallel design, high-fat western diet-fed, *n* = 7, blinding: single	500 *μ*M (339.41 mg·day^−1^) MitoQ given as *α*-cyclodextrin complex of the methanesulfonate salt for 14 weeks	Protein oxidation (PC), mitochondrial DNA in heart and liver samples
Miquel et al., 2014 [24]	Male and female transgenic ALS mice carrying the G93A-mutated human SOD1 strain, 90 days of age	Parallel design, ALS mouse model, *n* = 30, blinding: single	500 *μ*M (339.41 mg·day^−1^) MitoQ (as *β*-cyclodextrin complex) dissolved into drinking water, for 20 days	Protein oxidation (3-NT), reactive oxygen species metabolism (SOD) in brain, heart, liver, and skeletal muscle (quadriceps) samples
Ng et al., 2014 [25]	Transgenic nematode strain CL2006 (unc-54/Aβ1–42) and wild-type (N2), 4 days post hatch	Parallel design, *Caenorhabditis elegans* model of Alzheimer's disease, *n* = 40, blinding: double	1 *μ*M (0.68 mg·day^−1^) MitoQ stock solutions prepared in deionised water before being added to NGM agar 6 days post hatching	Protein oxidation (PC) of whole body (nematode) sample and oxidative damage (mtDNA) in mitochondria of nematodes
Ojano-Dirain et al., 2014 [35]	Albino male guinea pigs (250–350 g)	Parallel design, rats undergoing auditory brainstem response, *n* = 17, blinding: single	0.03 g·l^−1^ MitoQ (as *β*-cyclodextrin complex of the methane sulfonate salt) dissolved in drinking water for 90 days	Protein oxidation (PC, 3-NT), antioxidant enzymes (SO) in inner ear (organ of corti) samples
Parajuli et al., 2012 [22]	Male farm pigs (25–30 kg)	Parallel design, cold storage of the kidneys, *n* = 10, blinding: single	100 *μ*M (67.88 mg·day^−1^) MitoQ flushed through the kidneys then stored with 1 l Belzer solution for 2 days	Protein oxidation (3-NT), mitochondrial ROS production in kidney samples
Powell et al., 2015 [29]	Male Sprague-Dawley rats (250–350 g)	Parallel design, rat model haemorrhagic shock and reperfusion, *n* = 10, blinding: single	5 mg·kg^−1^ (1.50 mg·day^−1^) MitoQ given IV then following resuscitation at 20 mg·kg^−1^ IP	Lipid peroxidation (TBARs), antioxidant enzyme activity (GPx, CAT, and SOD) in liver sample
Rodriguez-Cuenca et al., 2010 [19]	Male C57BL/6 mice, 4–6 weeks of age	Parallel design, MitoQ supplemented in drinking water, *n* = 12, blinding: single	500 *μ*M (339.41 mg·day^−1^) MitoQ, (as a *β*-cyclodextrin complex of the methane sulfonate salt) dissolved in drinking water for 28 weeks	Protein oxidation (PC), oxidative damage (DNA) in heart and liver sample
Sakellariou et al., 2016 [20]	Male and female wild-type C57BL/6 mice, 24 months of age	Parallel design, CRM rodent diet-fed, *n* = 16, blinding: single	Mitoquinone mesylate, at 100 *μ*M (67.88 mg·day^−1^), (as a *β*-cyclodextrin complex) in drinking water for 15 weeks	Lipid peroxidation (8-OHdG), protein oxidation (3-NT), oxidative damage (DNA), mitochondrial membrane potential in skeletal muscle (anterior tibialis) sample
Shill et al., 2016 [33]	Males, 18–40 yr old, healthy	Parallel design, participants undergoing moderate exercise, *n* = 20, blinding: double	10 mg MitoQ pill daily with breakfast for 3 weeks	Lipid peroxidation (MDA, TBARs), oxidative damage (DNA) in blood samples
Supinski et al., 2009 [17]	Male and female rats (200–250 g) and mice (25–35 g)	Parallel design, *E. coli* model of sepsis, *n* = 8, blinding: single	500 *μ*M (339.41 mg·day^−1^) MitoQ injected IP with endotoxin for 2 days	Protein oxidation (PC) in heart samples
Wani et al., 2011 [32]	Male albino rats (100–150 g)	Parallel design, stress induced, *n* = 10, blinding: single	100 *μ*mol·kg^−1^·day^−1^ (67.88 mg·day^−1^) MitoQ dissolved in purified water for 12 weeks	Lipid peroxidation (MDA, TBARs, 8-OHdG), protein oxidation (PC), antioxidant enzymes (SOD) in brain sample
Xiao et al., 2017 [38]	Male diabetic C57BL/6 mice, 12 weeks old	Parallel design, 3-arm, *n* = 36, blinding: single	IP injection (5 mg twice wk^−1^) for 12 weeks	Mitochondrial membrane potential, Nrf2 in cultured kidney cells (human proximal tubular cell line, HK-2)

TBARs: thiobarbituric acid reactive substance; GPX: glutathione peroxidase; CAT: catalase; SOD: superoxide dismutase; PC: protein carbonyl; MDA: malondialdehyde; ROS: reactive oxygen species; Nrf2: NF-E2-related factor 2.

**Table 2:** Non-meta-analysed data table.

Outcome	Intervention			Control			Unit
Author, year	Mean	SD	*n*	Mean	SD	*n*	
*Lipid peroxidation*							
Lipid peroxidation							
McManus et al., 2011 [21]	1.2	0.5	6	4.9	1.96	6	*μ*M·mg protein^−1^
McManus et al., 2011 (dTPP) [21]	1.2	0.5	6	3.9	1.96	6	*μ*M·mg protein^−1^
8-Isoprostane							
Coudray et al., 2016 [31]	151	48	8	73	26	8	pg·mg protein^−1^
8-OHdG							
Wani et al., 2011 [32]	0.38	0.08	5	0.41	0.01	5	ng·ml^−1^
Lowes et al., 2008 [27]	16	3.5	10	23.5	4	6	ng·16 hr^−1^
8-OHdG (DNA)							
Sakellariou et al., 2016 [20]	2.7	0.2	5	2.8	0.1	5	ng·ml^−1^
TBARS							
Wani et al., 2011 [32]	168	44.9	5	172	56.1	5	nmol·*μ*g protein^−1^
MDA							
Maiti et al., 2017 [23]	0.47	0.24	6	0.47	0.24	6	nmol·mg protein^−1^
Powell et al., 2015 [29]	1.2	2.24	5	9	2.24	5	*μ*M MDA·mg tissue^−1^
Shill et al., 2016 [33]	7.7	2.53	10	9.1	0.9	10	*μ*M

*Antioxidant enzymes*							
CAT content							
Coudray et al., 2016 [31]	709	40	8	672	104	8	U^·^mg protein^−1^
CAT activity							
Powell et al., 2015 [29]	2	0.2	5	2.6	0.2	5	*μ*M·min^−1^
Powell et al., 2015 (dTPP) [29]	2	0.2	5	2.5	0.2	5	*μ*M·min^−1^
GPx activity							
Coudray et al., 2016 [31]	4.72	0.87	8	2.56	0.26	8	
Sakellariou et al., 2016 [20]	0.9	0.2	5				Ratio to control
Powell et al., 2015 [29]	38	7	5	20	7	5	*μ*M·min^−1^·ml^−1^
Powell et al., 2015 (dTPP) [29]	38	7	5	30	7	5	*μ*M·min^−1^·ml^−1^
MnSOD expression							
Coudray et al., 2014 [31]	2.47	0.32	8	2.56	0.26	8	U·mg protein^−1^
Ojano-Dirain et al., 2014 [35]	1.3^∗^	0.18	9	1	0.14	8	Protein expression relative to control
Ojano-Dirain et al., 2014 (dTPP) [35]	1.3	0.18	9	1.08	0.24	9	Protein expression relative to control
Wani et al., 2011 [32]	4.9	0.64	5	4.62	0.5	5	U·mg protein^−1^

*ROS*							
Coudray at al., 2016 [31]	650	145	8	580	147	8	pM H_2_O_2_·min^−1^·mg protein^−1^
Ng et al., 2014 [25]	6	2.6	3	7	3.5	3	RFU·min^−1^
Ng et al., 2014 (dTPP) [25]	6	2.6	3	6	2.6	3	RFU min^−1^

*Mitochondrial DNA damage*							
mtDNA damage amplification							
Dare et al., 2015 [28] (0 h reperfusion)	1.1	0.2	5	0.75	0.6	4	Relative amplification
Dare et al., 2015 [28] (1 h reperfusion)	0.97	0.16	4	1.05	0.1	4	Relative amplification
Dare et a., 2015 (24reperfusion) [28]	0.8	0.3	4	0.2	0.1	4	Relative amplification

*Gene-related outcomes*							
Nrf2							
Coudray et al., 2016 [31]	1.1	0.25	8	1.23	0.31	8	Ratio to control
Lowes et al., 2008 [27]	0.8	1.1	12	0.25	0.5	12	Intensity
SOD2 gene exp							
Sakellariou et al., 2016 [20]	1	0.04	5				Ratio to control
SOD gene exp							
Powell et al., 2015 [29]	6	1.34	5	5.5	0.67	5	U·ml^−1^
Powell et al., 2015 (dTPP) [29]	6	1.34	5	6.5	1.57	5	U·ml^−1^

TBARs: thiobarbituric acid reactive substance; GPX: glutathione peroxidase; CAT: catalase; SOD: superoxide dismutase; PC: protein carbonyl; MDA: malondialdehyde; ROS: reactive oxygen species.
